# Dentin matrix protein-1 promoted osteogenic differentiation of valvular interstitial cells via MAPK signal pathway during aortic valve calcification

**DOI:** 10.1007/s11626-025-01101-7

**Published:** 2025-10-13

**Authors:** Jinjie Chen, Yefan Jiang, Si Chen, Junxiang Liu, Wenjing Zhang, Yixuan Wang, Geng Li

**Affiliations:** 1 Department of Cardiovascular Surgery, Union Hospital, Tongji Medical College, Huazhong University of Science and Technology, Wuhan, 430022 China; 2 Department of Cardiovascular Surgery, The First Affiliated Hospital of Nanjing Medical University, Nanjing, 210000 China; 3 Department of Ultrasound Medicine, Union Hospital, Tongji Medical College, Huazhong University of Science and Technology, Wuhan, 430022 China

**Keywords:** Dentin matrix protein-1, Valvular interstitial cells, Integrin receptors, MAPK signaling pathway, Calcific aortic valve disease

## Abstract

Valvular interstitial cells (VICs) are integral to the progression of calcific aortic valve disease (CAVD). Dentin matrix protein-1 (DMP-1), a member of the Sibling family protein, is implicated in the calcification process. This study aims to investigate the role and mechanisms of DMP-1 in the osteogenic differentiation of VICs. Between April 2018 and December 2018, aortic valve tissues were collected from 14 patients undergoing aortic valve replacement or heart transplantation. DMP-1 expression was quantified in calcified valves versus normal controls. An in vitro model of VICs’ osteogenic differentiation was established to study the regulatory mechanism of DMP-1 on valvular calcification using immunoblot, immunohistochemistry, immunofluorescence, etc. The expression of DMP-1 was significantly increased in the calcified aortic valves patients (*P* < 0.01). DMP-1, both short and long arginine-glycine-aspartic acid (RGD) peptides, induced the osteogenic differentiation in VICs, an effect that was inhibited by an integrin αvβ3 antagonist (*P* < 0.05). Furthermore, the expression levels of RAF, RAS, MEK, and phosphorylated ERK1/2 were significantly elevated in VICs upon stimulation of DMP-1 (*P* < 0.05). DMP-1 is involved in the progression of valvular calcification and promotes the osteogenic differentiation of VICs via the integrin αvβ3 receptor. The combination of DMP-1 and integrin αvβ3 via its RGD domain activates the MAPK signaling pathway, leading to enhanced osteogenic gene expression in VICs. Clinical trial number: not applicable.

**Supplementary Information:**

The online version contains supplementary material available at 10.1007/s11626-025-01101-7.

## Introduction

Calcific aortic valve disease (CAVD) is the most common cardiovascular disease, with its incidence arising due to the aging of the population (Lindman *et al.* 2016; Mensah *et al.* 2017). At present, surgical intervention remains the sole effective treatment for CAVD; however, no effective drugs are available to restrict the development of cardiac valve degeneration (Everett *et al.* 2018). Therefore, clarifying the mechanism of cardiac valve degeneration is important for identifying potential drug targets to prevent CAVD progression.

Valvular interstitial cells (VICs) are believed to play an important role in the development of CAVD (Rutkovskiy *et al.* 2017; Yang *et al.* 2022). Disruptions in the microenvironment are closely associated with the migration, differentiation, adhesion, and apoptosis of VICs (Lin and Bissell 1993). Recent studies have revealed that VICs can undergo osteogenic differentiation upon stimulation by TGF-β or BMP-2 (Kaden *et al.* 2004; Liu *et al.* 2018). In calcified aortic valves, osteogenic VICs expressing bone markers such as runt-related transcription factor 2 (RUNX2) contribute to the formation of calcification nodules (Dharmarajan *et al.* 2021; Yan *et al.* 2022). Therefore, investigating the mechanism of VICs’ osteogenic differentiation is important for providing effective therapeutics for CAVD.

During the progression of valvular calcification, significant alterations in the extracellular matrix (ECM) organization and composition, such as the disarrangement of collagen fibers (Vadana *et al*. 2020; Di Vito *et al*. 2021; Weber *et al*. 2021). The ECM contains numerous exocrine proteins, among which Siblings family proteins are integral to osteogenesis (Li *et al*. 2019). Dentin matrix protein-1 (DMP-1), a member of the Siblings proteins, is abundant in the ECM of bones and teeth (Qin *et al*. 2007). DMP-1 features an arginine-glycine-aspartic acid (RGD) domain at its C-terminal, which can bind to integrin receptors and mediate cell migration and adhesion (He and George 2004; Ngai *et al*. 2018). Integrin αvβ3 is a dimer receptor, which consists of αv and β3 subunits. The combination of RGD peptide and β3 subunit can activate intracellular signaling pathways and promote the expression of target genes (Benton *et al*. 2009). Understanding the function of DMP-1 in valvular calcification is helpful to discover new therapeutic targets.

In the present study, we collected the aortic valve samples to investigate the role of DMP-1 in valvular calcification and constructed a VIC calcification model to study the mechanism of DMP-1 in the progression of CAVD. The results of our study may provide insights into the pathogenesis of CAVD.

## Material and methods

### Patient recruitment and sample collection

Calcified aortic valves and human serum samples were obtained from CAVD patients who were undergoing aortic valve replacement surgery at the Department of Cardiovascular Surgery of Wuhan Union Hospital between April 2018 and December 2018. A total of 14 patients were included in the study, among whom 11 had calcified aortic valves and 3 patients with non-calcified valves (confirmed by echocardiographic assessment) served as controls. Patients with a history of rheumatic disease, infective endocarditis, bicuspid aortic valves, or any other significant valvular disease were excluded. Non-calcified aortic valves and serum were obtained from patients undergoing heart transplantation. The study protocol was in compliance with the Declaration of Helsinki and was approved by the Ethics Review Board of Union Hospital and Tongji Medical College (Approval No.: [UHCT-IEC-SOP-016–03-01]). All patients provided written informed consent prior to inclusion in the study.

### Antibodies

Rabbit polyclonal anti-human RUNX2 (Abcam, Cambridge, UK), rabbit polyclonal anti-human DMP-1 (Abcam, UK), rabbit polyclonal anti-human integrin αv (Abcam), and rabbit polyclonal anti-human integrin β3 (Santa) were used for immunohistochemical assay. Anti-RUNX2 (Santa, USA), anti-DMP-1 (Abcam), anti-integrin αv (Abcam), anti-ALP (R&D, Minneapolis, MN), anti-integrin β3 (Santa), anti-RAS (CST, Danvers, MN), anti-MEK (CST), anti-ERK (CST), and anti-p-ERK (CST) were used for immunoblotting assay. Anti-α-SMA (Boster, Wuhan, China), anti-vimentin (Boster), DAPI (BioFroxx GmbH, Einhausen, Germany), anti-human integrin β3 (Santa), and anti-human DMP-1 (Abcam) were used for immunofluorescence.

### Isolation and culture of human VIC

Human VICs were isolated from non-calcified aortic valves obtained from patients undergoing heart transplantation, as previously described (Yang *et al.* 2022). Briefly, valve leaflets were digested in a 1 mg/mL solution of type I collagenase for 30 min, followed by scraping to remove valvular endothelial cells. Then, the valves were subjected to a fresh collagenase solution for 6 h, after which the cells were centrifuged and resuspended. The VICs were cultured in Dulbecco’s modified Eagle medium containing 100U/mL penicillin G, 100 U/mL streptomycin, and 10% fetal bovine serum. The cells were passaged 2 to 4 generations for the experiments. Mycoplasma contamination was assessed using a mycoplasma detection kit (Invitrogen, Carlsbad, CA). The expression of Vimentin and α-SMA in cultured cells was detected by fluorescence immunoimaging, verifying the successful isolation of aortic interstitial cells.

### In vitro osteogenic differentiation of VIC

The calcification of VICs was induced by osteogenic medium (Dulbecco’s modified Eagle medium containing 5% fetal bovine serum, 50 mg/mL ascorbic acid, 100 nmol/L dexamethasone, and 10 mmol/L β-glycerophosphoric acid). To investigate the effect of DMP-1 on the osteogenic differentiation of VICs, 500 ng/mL DMP-1 was added to the DMEM medium. After 3 d of incubation, protein and mRNA were extracted from VICs for immunoblot and qPCR assays respectively. After 21 days of incubation, the Alizarin Red staining was used for calcium deposition quantification.

### Immunohistochemical assay

The immunohistochemical assays were performed on paraffin sections of aortic valve tissues as previously described (Dharmarajan *et al.* 2021). After antigen retrieval, the deparaffinized sections were incubated with primary antibody overnight at 4°C, followed by incubation with the appropriate secondary antibody. The DAB horseradish peroxidase color development kit (Beyotime, Shanghai, China) was utilized to visualize the staining signal. The expression of the target gene was analyzed using Image-Pro Plus (Media Cybernetics, Inc, Rockville, MD).

### Alizarin Red stain

Aortic valve excised during surgery was placed in a container filled with cold sodium chloride physiological solution. One cusp was fixed in 4% paraformaldehyde, dehydrated, and subsequently embedded in paraffin. VICs were washed twice with PBS and fixed in 4% paraformaldehyde for 15 min. Alizarin Red stain was performed on paraffin sections of aortic valve tissues or VICs fixed with 4% paraformaldehyde. After incubation with 0.2% Alizarin Red solution for 30 min, excess dye was removed by washing with distilled water. The images were captured using an optical microscope (Olympus, Tokyo, Japan), and the calcification nodules appeared orange/red.

### Enzyme-linked immunosorbent assay

Blood serum was collected before the operation. Concentrations of DMP-1 in blood serum and culture medium were measured by enzyme-linked immunosorbent assay (Abcam) according to the manufacturers’ protocol.

### Immunofluorescence

Frozen sections of aortic valves and VICs were subjected to immunofluorescence assay. Immerse valves or cells with 0.1% Triton X-100 for 5–10 min and wash with PBS for 3 times. Then, sections were blocked with 5% BSA at room temperature for 30 min and incubated with primary antibody at 4°C overnight. After washing with PBS for 3 times, sections were incubated with second antibody at room temperature for 30 min in the dark. After further PBS washes, nuclei were stained with DAPI at room temperature for 15 min in the dark. Finally, coverslips were mounted with glycerinum, and images were captured using a fluorescence microscope (Nikon, Tokyo, Japan).

### Immunoblot analysis

Aortic valves were dissected into small pieces and smashed with liquid nitrogen. Proteins were extracted and lysed in RIPA Lysis and Extraction Buffer (Beyotime) containing proteases and phosphatase inhibitor cocktail (ThermoFisher, Waltham, MA) for 30 min on ice. VICs Protein extracts were prepared by washing cells with cold PBS and lysing in RIPA Buffer containing proteases and phosphatase inhibitor for 10 min on ice. After centrifugation at 12,000 g for 10 min at 4°C, supernatants were then transferred to new Eppendorf tubes, and protein concentrations were measured using the BCA method. The expression of target protein was detected using specific antibodies following standard procedures.

### RNA isolation, cDNA synthesis, and qPCR

Aortic valves were dissected into pieces and homogenized using liquid nitrogen. Total RNA from the valves and VICs was prepared using Trizol reagent (ThermoFisher). The RNA-containing supernatants were further purified with the Direct-zol RNA MicroPrep Kit (Zymo Research, Irvine, CA), and the concentration of RNA was measured using a NanoDrop (ThermoFisher). 0.1–1 µg of total RNA was reverse transcribed using the cDNA Synthesis Kit (ThermoFisher) according to the manufacturers’ protocol. The cDNA was diluted tenfold for subsequent qPCR analysis conducted on an ABI StepOnePlus System (Applied Biosystems, Waltham, MA). Primers for qPCR (Table 1) were designed using Primer 5.0 software and synthesized by GenScript Co., Ltd (Nanjing, China). Relative gene expression levels were normalized to GAPDH expression. The 2-ΔΔCT calculation method was used for analyzing the data.

**Table 1.** Primers for qPCR

Primers	Sequence
ALP forward	5′-TCAAGCCAAGACACAAGCAC-3′
ALP reverse	5′-TTCCACCAGCAAGAAGAAGC-3′
RUNX2 forward	5′-TGGCAGGGTCTCTTGTTTGA-3′
RUNX2 reverse	5′-TGAAGAGACTCAGGCGCTAC-3′
DMP-1 forward	5′-CTCAAGATTCAGGTGGCAGC-3′
DMP-1 reverse	5′-AGTCACCCTTGCTGTCTCTC-3′
GAPDH forward	5′-GGAGCGAGATCCCTCCAAAAT-3′
GAPDH reverse	5′-GGCTGTTGTCATACTTCTCATGG-3′

### Statistical analysis

All results were analyzed by SPSS 22.0 software (SPSS lnc, Chicago, IL). Pearson’s correlation coefficient was calculated to assess the relationships between continuous variables. Student’s *t*-test was used for comparisons between two groups, and one-way ANOVA was utilized for comparisons among more than three groups. Data are expressed as mean ± SE. *P*-value < 0.05 was considered statistically significant. Densitometric analysis of Western blot bands was performed using ImageJ software. The relative expression level of each target protein was normalized to the corresponding GAPDH band intensity.

## Results

### Upregulated expression of DMP-1 and integrin β3 was detected in calcified aortic valves

**Figure 1.**
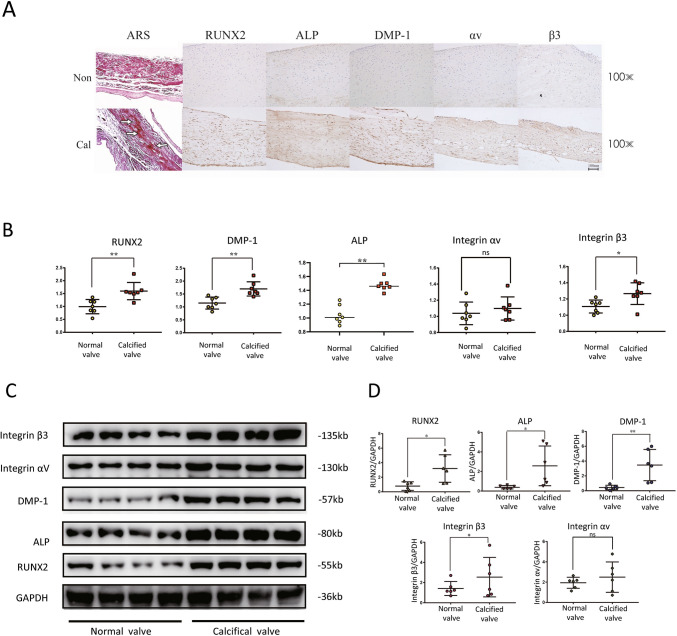
The expression of DMP-1 and integrin β3 increase in calcified aortic valve. (*A*) Immunohistochemistry of normal valve or calcified valve stained with RUNX2, DMP-1, integrin β3, and integrin αv antibodies. (*B*) Comparison of staining intensity between groups in immunohistochemical images. Data are mean ± SD (*n* = 7). ns, not significant; *P < 0.05; **P < 0.01 (unpaired two-tailed Student’s *t*-test). (*C*) Immunoblot analysis of integrin αv, integrin β3, DMP-1, ALP, and RUNX2 expression in normal valve and calcified valve. (*D*) The *dot plots* displayed the quantification of integrin αv, integrin β3, DMP-1, ALP, and Runx2 level shown in *panel C*. Data are mean ± SD (*n* = 6). ns not significant; *P < 0.05; **P < 0.01.

Calcified and normal aortic valves were obtained from patients, and the baseline characteristics of all participants are shown in Table 2. No significant differences were observed in demographic properties, serum LDL concentration, serum creatine, ejection fraction between control and CAVD groups. Alizarin Red staining demonstrated that calcified valves had significantly more calcification nodules compared with normal valves (Fig. 1*A*). Elevated expression of RUNX2, the osteogenic markers, was detected in calcified valves (Fig. 1*A*, *B*). Meanwhile, the expression of ALP, which correlates positively with calcification, was significantly elevated in calcified aortic valves (Fig. 1*C*, *D*). Immunohistochemical staining demonstrated that DMP-1 and integrin β3 were significantly increased in calcific valves, with no significant change in the expression of integrin αv (Fig. 1*A*, *B*). Immunoblot assays further confirmed the upregulated expression of DMP-1 and integrin β3 in calcified aortic valves (Fig. 1*C*, *D*). These data indicated that DMP-1 and integrin β3 might involve in valvular calcification.

**Table 2.** Baseline characteristics data of all participants enrolled in this study

Parameters	CAVD group (*n* = 7)	Control group (*n* = 7)	*P*-value	*t*-value
Age (years)	50.14 ± 1.85	48.57 ± 11.69	0.822	1.927
Male, *n* (%)	6 (85.7)	4 (57.1)	/	/
LDL-C (mmol/L)	2.02 ± 0.82	2.32 ± 0.87	0.514	1.578
Creatine (μmol/L)	68.23 ± 19.42	68.89 ± 16.23	0.946	0.1244
EF (%)	37.86 ± 22.93	50.43 ± 14.41	0.243	0.8794

*LDL-C* low-density lipoprotein-cholesterol, *EF* ejection fraction

### The expression of DMP-1 and integrin β3 increased in osteoblastic VIC

To further identify the role of DMP-1 in valvular calcification, primary aortic VICs were successfully isolated, as cells were vimentin and α-SMA double positive in immunofluorescence assay (Supplementary Fig.  1*A*).

**Figure 2.**
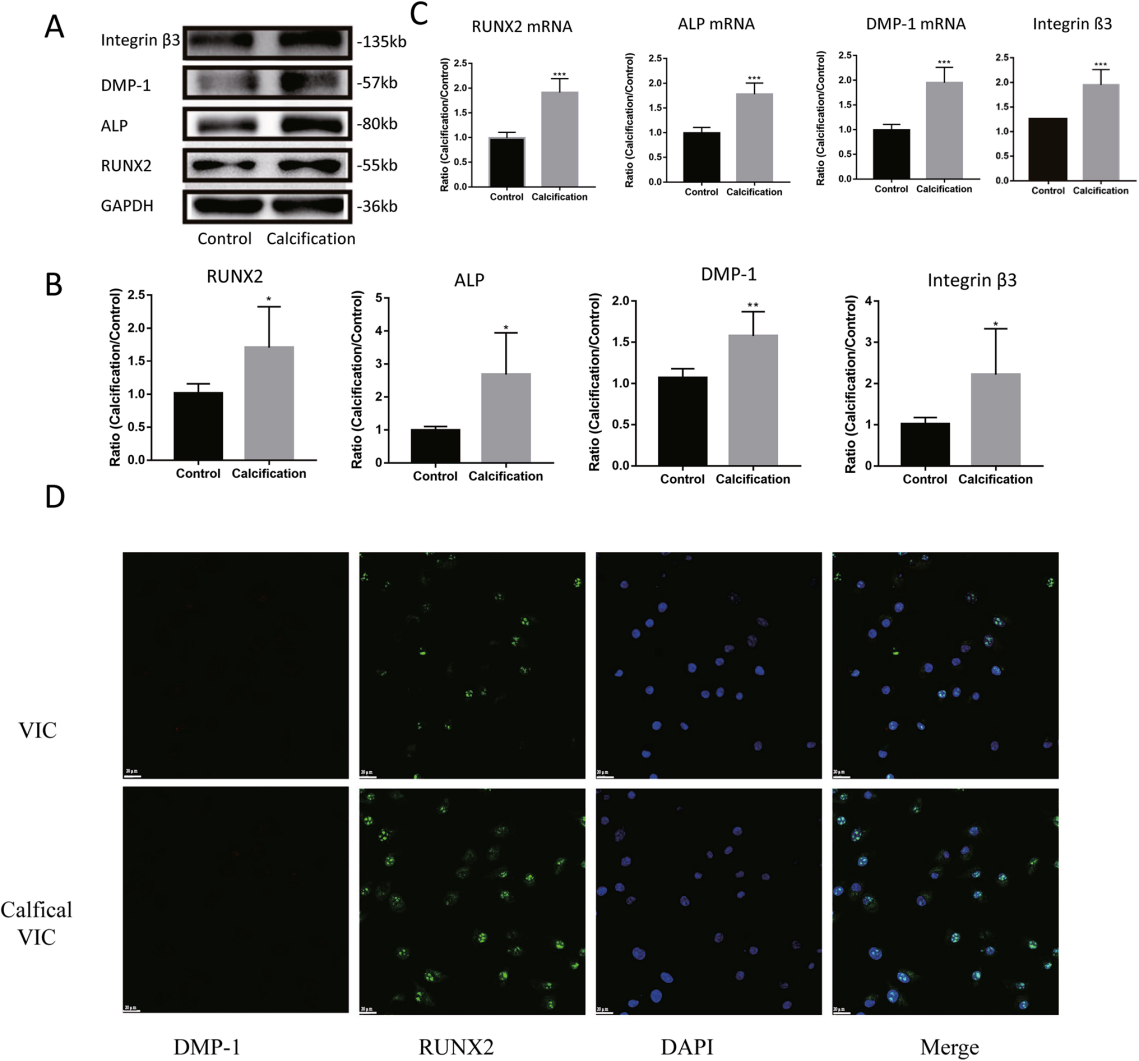
The expression of DMP-1 and integrin αvβ3 increase in the osteoblastic VICs. (*A*) Immunoblot analysis of integrin β3, integrin αv, DMP-1, ALP, RUNX2, and GAPDH expression in normal or calcific VICs. (*B*) Comparison of integrin β3, integrin αv, DMP-1, ALP, and RUNX2 expression between the control and calcification groups. (*C*) Comparison of RUNX2, integrin ß3, and DMP-1 mRNA levels between normal and VICs. (*D*) Immunofluorescence staining for DMP-1 (*red*), RUNX2 (*green*), and DAPI (*blue*) in VICs. Data are mean ± SD (*n* = 5). *P < 0.05; **P < 0.01; ***P < 0.001.

Osteoblastic VICs were successfully induced in osteogenic medium as calcification nodules showing in Alizarin Red staining (Supplementary Fig. 1*B*). Immunoblot analysis demonstrated that the expression of RUNX2, ALP, DMP-1, and integrin β3 increased in osteoblastic VICs (Fig. 2*A*, *B*). Besides, the mRNA levels of DMP-1, RUNX2, and ALP also elevated with the osteogenic differentiation of VICs, and immunofluorescence staining demonstrated that DMP-1 was mainly located within cells (Fig. 2*D*). Thus, DMP-1 could be induced in VICs and participate in osteogenic differentiation.

### The expression of DMP-1 was correlated with integrin β3 in aortic valves

**Figure 3.**
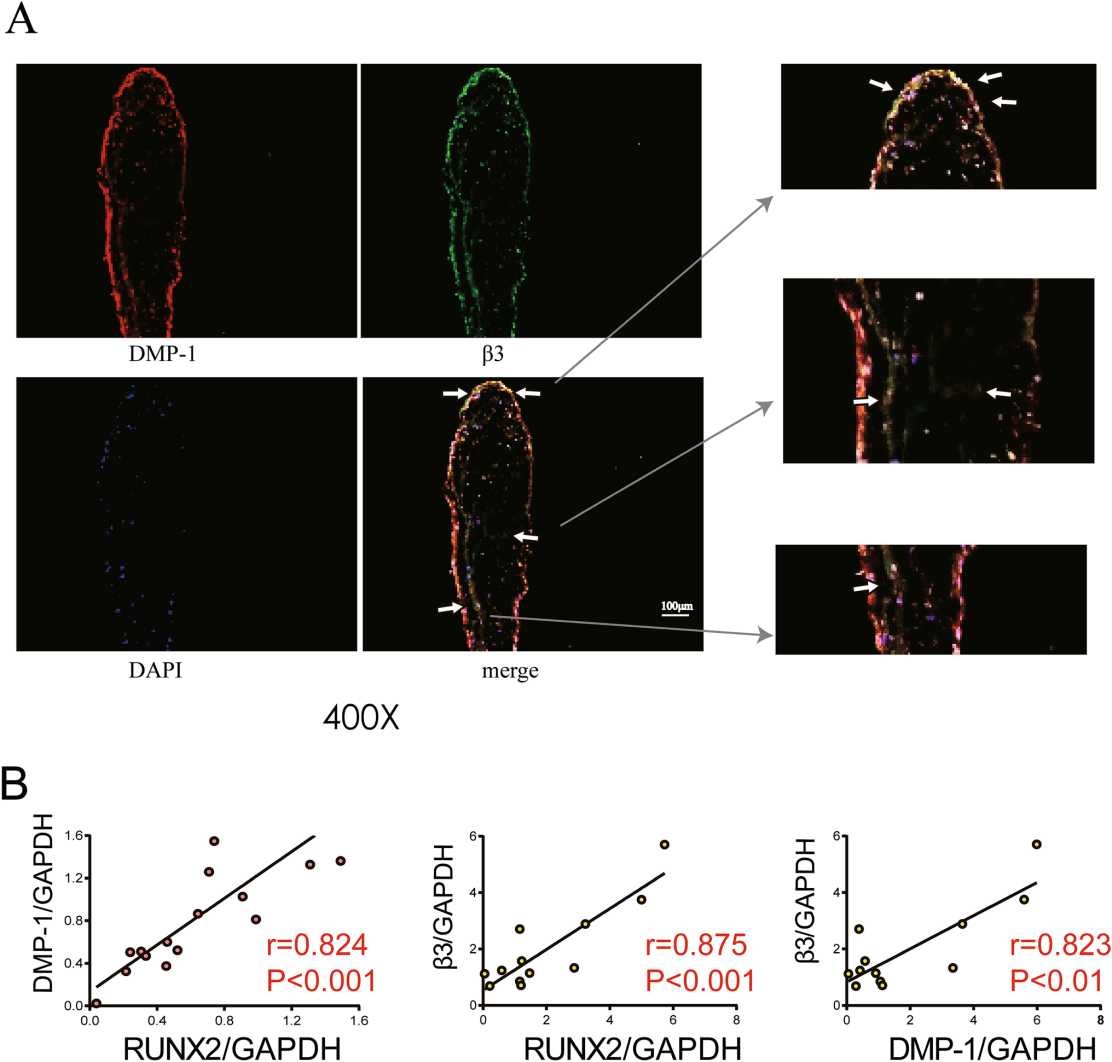
The expression of DMP-1 is correlated with integrin β3 in calcified aortic valve. (*A*) Immunofluorescence staining of human aortic valve tissue sections showing the localization of DMP-1 (*red*), integrin β3 (*green*), and nuclei (DAPI, *blue*). (*B*) Pearson’s correlation analysis of DMP-1, integrin β3, and RUNX2 expression levels based on densitometric quantification of Western blot results from aortic valve tissues (*n* = 10). Each *dot* represents an individual tissue sample. R, correlation coefficient. *P* < 0.05 was considered statistically significant.

The integrin receptor–mediated intracellular signaling can activate the expression of osteogenic genes. Immunofluorescence staining assays were performed and results demonstrated that DMP-1 could be detected in integrin β3 positive cells, indicating the combination of DMP-1 and integrin β3 (Fig. 3*A*). According to the linear correlation analysis of the result of the Western blot of integrin β3, DMP-1, RUNX2 expression in normal valve and calcified valve, we find the protein level of DMP-1 was positively correlated with integrin β3, both of which were associated with the expression of RUNX2 (Fig. 3*B*). Thus, the interaction between DMP-1 and integrin β3 might participate in the calcification of the aortic valve.

### DMP-1 could induce the osteogenic differentiation of VIC

**Figure 4.**
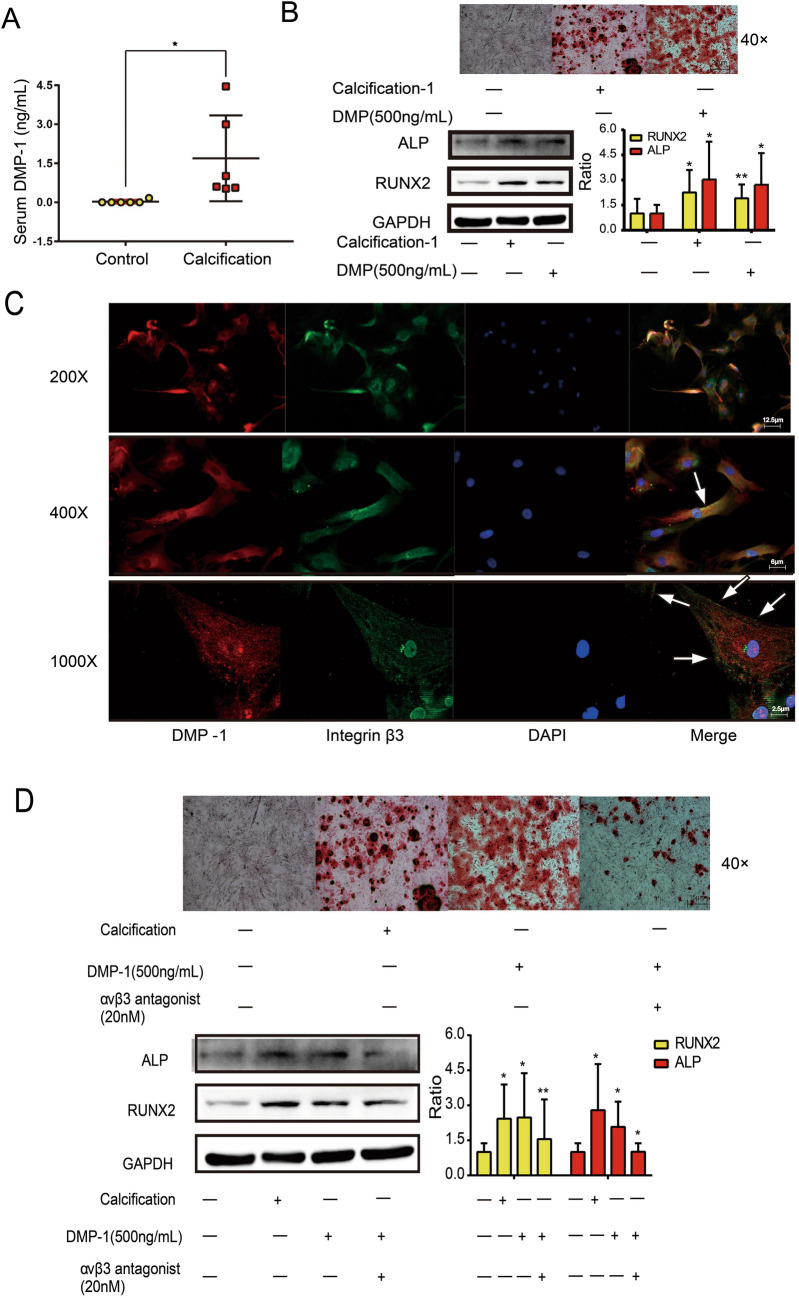
The combination of DMP-1 and integrin αvβ3 contributes to the calcification of VICs. (*A*) The concentration of DMP-1 in serum from patients with normal aortic valve or calcific valve. Data are mean ± SD (*n* = 6). *P < 0.05 (unpaired two-tailed Student’s *t*-test). (*B*) Alizarin Red S staining was used to assess calcium nodule formation in VICs following different treatments. The *red color* indicates areas of calcium deposition, representing osteogenic differentiation. Immunoblot analysis of ALP and RUNX2 expression in VICs in calcification, DMP-1 treatment, and control groups. Data are mean ± SD (*n* = 5). *P < 0.05; **P < 0.01 (unpaired two-tailed Student’s *t*-test). (*C*) Immunofluorescence staining of DMP-1 (*red*), integrin β3 (*green*), and DAPI (*blue*) in VICs. The *arrows* point to the co-expression of DMP-1 and integrin β3. (*D*) Western blot and Alizarin Red analysis of ALP and RUNX2 expression in calcification, DMP-1, αvβ3 receptor blocker, and control groups. Data are mean ± SD (*n* = 5). *P < 0.05; **P < 0.01.

We detected the serum DMP-1 concentration, and results demonstrated that DMP-1 was significantly higher in the serum of CAVD patients (Fig. 4*A*). We cultured VICs with stimulation of DMP-1, and DMP-1 could also induce the osteogenic differentiation of VICs, which was comparable with osteogenic medium. As shown in Fig. 4*C*, the arrows point to the co-localization of DMP-1 and integrin β3 in osteoblastic VICs. Immunofluorescence assay demonstrated that DMP-1 co-localized with integrin β3 in osteoblastic VICs, and the addition of integrin αvβ3 antagonist could inhibit the osteogenic differentiation of VICs (Fig. 4*D*). These data indicated that the combination of DMP-1 and integrin β3 plays an important role in the induction of osteoblastic VICs.

### RGD peptide induced the osteogenic differentiation of VICs via integrin αvβ3

**Figure 5.**
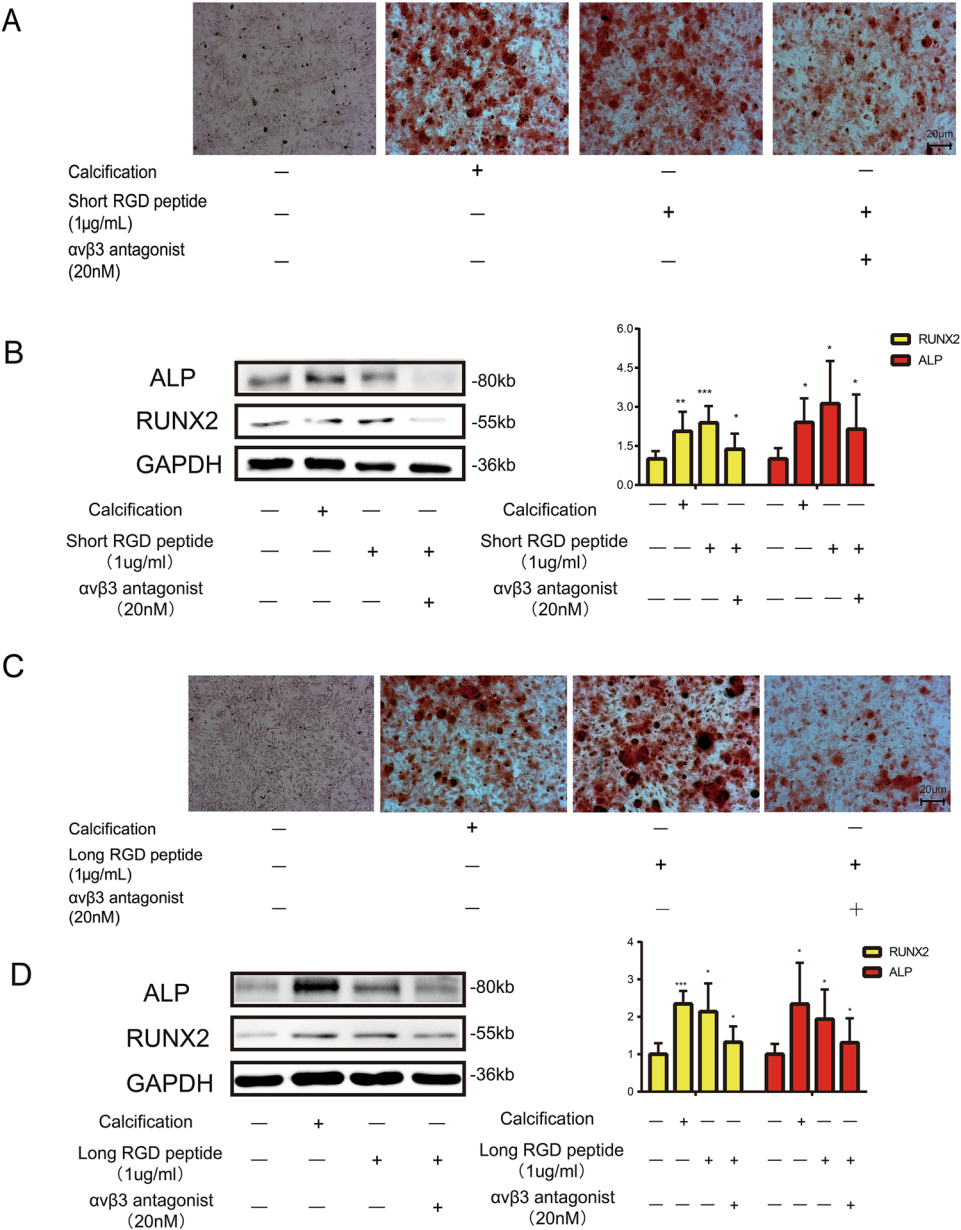
DMP-1 combines with integrin αvβ3 via RGD domain. (*A*) Alizarin Red stain of VICs in calcification, short-chain RGD treatment with or without integrin αvβ3 receptor blocker and control groups. (*B*) Immunoblot analysis of ALP and RUNX2 expression in calcification, short-chain RGD treatment with or without integrin αvβ3 receptor blocker and control groups. Alizarin Red S staining of VICs after 21 d of culture under the following conditions: control group, calcification-inducing medium alone, long-chain RGD peptide treatment without αvβ3 antagonist (−), and long-chain RGD peptide treatment with αvβ3 antagonist (+). *Red staining* indicates calcium deposition, reflecting the degree of calcification. (*D*) Immunoblot analysis of ALP and RUNX2 expression in calcification, long-chain RGD with or without integrin αvβ3 receptor blocker and control groups. Data are mean ± SD (*n* = 5). *P < 0.05; **P < 0.01; ***P < 0.001.

There is an integrin-binding site located at the C-terminal of DMP-1, and the N-terminal of DMP-1 is associated with its combination with type I collagen. DMP-1 can bind to integrin via RGD domain in the dissociated state or type I collagen binding state (Pawade *et al.* 2015). So we use the exogenous recombination RGD peptide to stimulate VICs to confirm the function of RGD domain. The experiment flow chart is shown in the figures (Supplementary Fig. 2). Short RGD peptide, which was soluble in culture medium due to the lack of N-terminal domain, could induce the osteogenic differentiation of VICs (Fig. 5*A*). Immunoblot analysis demonstrated that the expression of ALP and RUNX2 elevated with the stimulation of short RGD peptide (Fig. 5*B*). The osteogenic effect of short RGD peptide could be inhibited by integrin αvβ3 antagonist (Selleck, S7834) (Fig. 5*A*, *B*). Long RGD peptide, which could adhere to culture dish via its N-terminal, could also induce the osteogenic differentiation of VICs, and such effect was inhibited by integrin αvβ3 antagonist (Fig. 5*C*, *D*). These data indicated that the binding of DMP-1 with integrin αvβ3 via RGD domain contributed to the osteogenic differentiation of VICs.

### DMP-1 induced the osteogenic differentiation of VICs through the MAPK pathway

**Figure 6.**
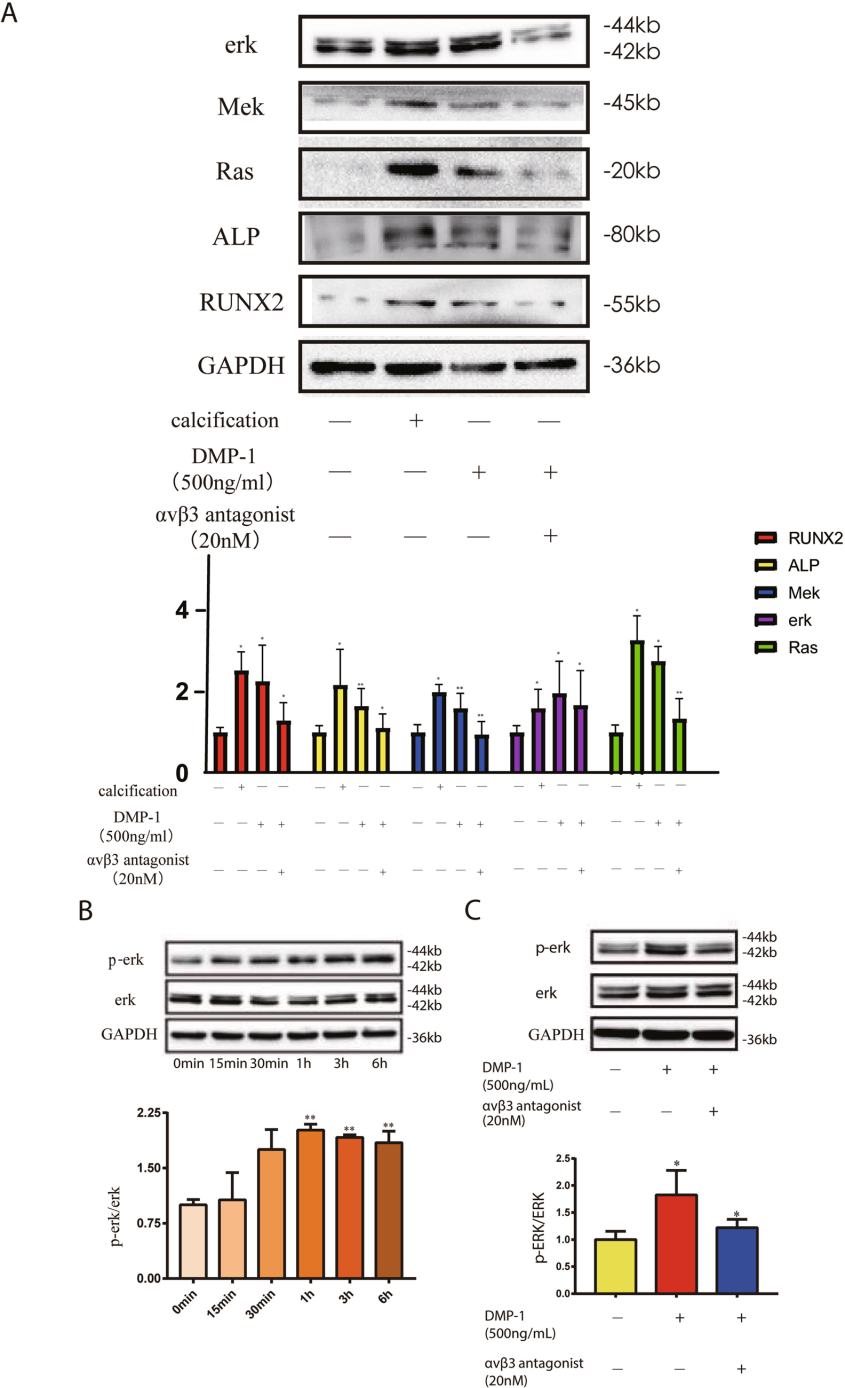
The combination of DMP-1 and integrin αvβ3 activates MAPK signaling pathway. (*A*) Immunoblot analysis of ERK, Mek, Ras, ALP, and RUNX2 expression in calcification, DMP-1, αvβ3 receptor blocker, and control groups. (*B*) Immunoblot analysis of p-ERK and erk in VICs stimulated with DMP-1 for different times. (*C*) Immunoblot analysis of p-ERK and erk in DMP-1 stimulated VICs with or without αvβ3 receptor blocker treatment. Data are mean ± SD (*n* = 5). *P < 0.05; **P < 0.01; ***P < 0.001.

Previous studies revealed that the MAPK pathway is involved in aortic valve calcification (Yang *et al.* 2020). Activation of RAF, RAS, and MEK can lead to the phosphorylation of ERK1/2, with the nuclear transportation of p-ERK1/2 regulating target gene expression. Immunoblot assays showed similar effects of DMP-1 and osteogenic medium on the osteogenic differentiation of VICs. The expression of RAF, RAS, MEK, ALP, and RUNX2 increased in the DMP-1 and calcification groups, and the upregulated expression of these genes could be inhibited by an integrin αvβ3 antagonist (Fig. 6*A*). We further stimulated VICs with DMP-1 for different times; the level of p-ERK started to increase at 30 min and peaked at 1 h (Fig. 6*B*). The treatment with the integrin αvβ3 antagonist could inhibit the elevation of p-ERK (Fig. 6*C*).

### Endogenous DMP-1 and integrin αvβ3 jointly contribute to osteogenic differentiation in VICs

**Figure 7.**
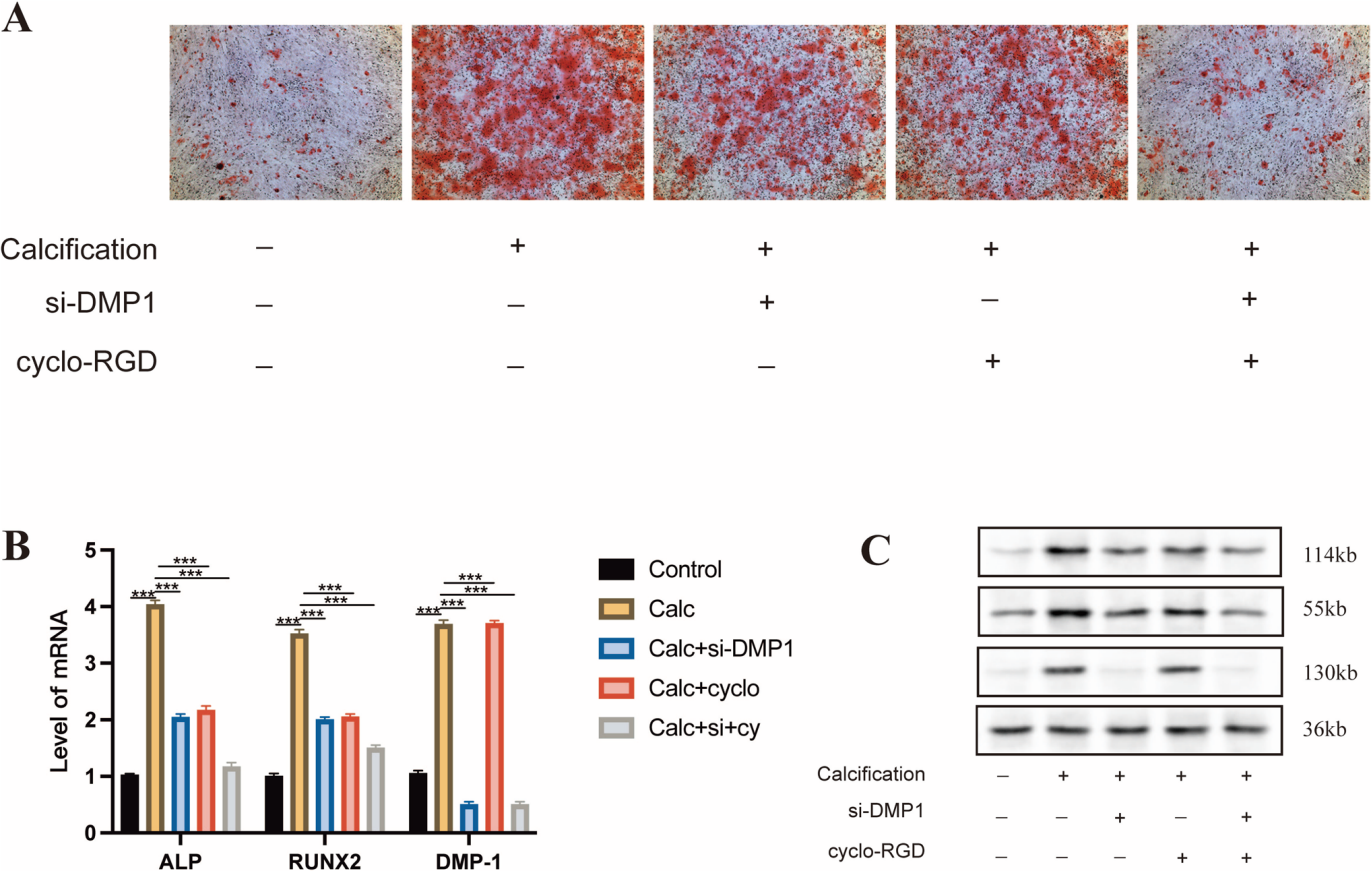
Endogenous DMP-1 and integrin αvβ3 jointly regulate osteogenic differentiation in VICs. (*A*) Schematic grouping of VICs cultured under calcification-inducing conditions with or without siRNA targeting DMP-1 and/or αvβ3 antagonist (cyclo-RGD). (*B*) Alizarin Red staining was used to assess the degree of calcium nodule formation in each group after 21 d of osteogenic induction. (*C*) Protein levels of ALP, RUNX2, and DMP-1 were measured by Western blot on day 7. Both DMP-1 knockdown and αvβ3 inhibition reduced ALP and RUNX2 expression, while combined treatment resulted in further suppression. GAPDH served as the internal control. Data represent mean ± SD (*n* = 3); *P < 0.05; **P < 0.01; ***P < 0.001.

**Figure 8.**
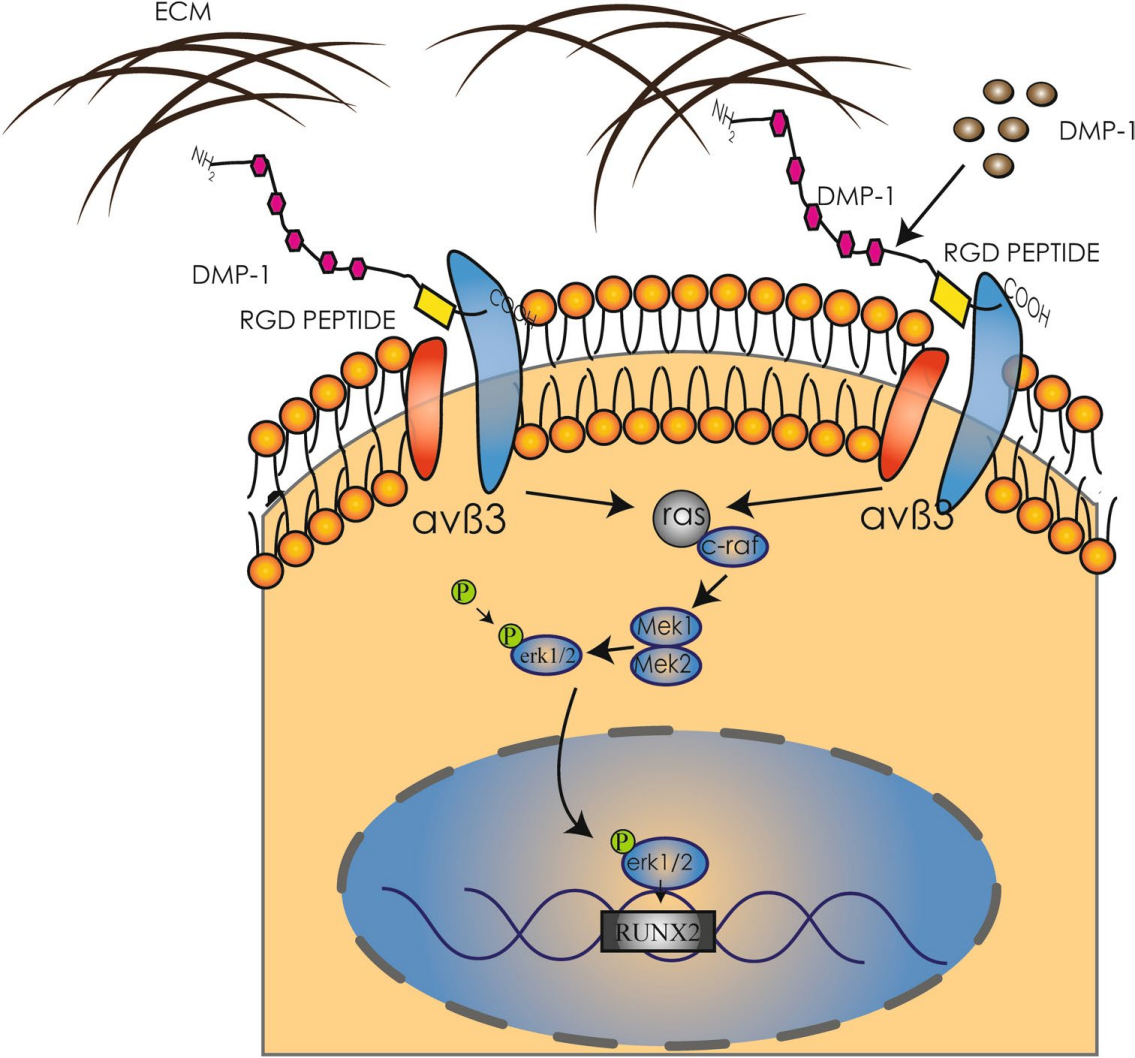
Diagram of DMP-1-mediated VIC calcification.

To investigate the involvement of endogenous DMP-1 and integrin αvβ3 in VIC calcification, we performed knockdown of DMP-1 using siRNA and simultaneously inhibited integrin αvβ3 activity with the specific antagonist cyclo-RGD under calcification-inducing conditions (Fig. 7). Western blot analysis revealed that the expression levels of osteogenic markers ALP and RUNX2 were significantly increased in calcified VICs. However, both DMP-1 knockdown and αvβ3 inhibition individually reduced these protein levels, and the combined treatment showed an even greater suppressive effect (Fig. 8*C*). These results suggest that DMP-1 and integrin αvβ3 play non-redundant but complementary roles in promoting osteogenic differentiation of VICs. The enhanced inhibition observed with dual intervention implies potential crosstalk or synergy between DMP-1 signaling and integrin αvβ3–mediated pathways in the progression of valve calcification. Based on these data, DMP-1 combined with integrin αvβ3 via its RGD domain and activated the MAPK signaling pathway; then, the translocation of p-ERK triggers the osteogenic gene expression, resulting in the induction of osteoblastic differentiation of VICs (Fig. 8).

## Discussion

The aortic valve is primarily composed of valvular endothelial cells (VECs) and VICs. VECs are located on the valve surface, and calcified nodules predominantly form in the fibrous layer, which is rich in VICs (Di Vito *et al*. 2021). Inflammation, mechanical stimulation, and other factors can injure VECs, leading to the exposure of ECM beneath the endothelial cells (Dweck *et al.* 2012). The infiltration of inflammatory cells in the valvular fibrous layer disrupts the local microenvironment (Lu *et al.* 2022). Previous studies demonstrated that large deposition of osteogenic protein, such as BMP-2 and BSP-2, could promote osteogenic differentiation of VICs (Kaden *et al.* 2004; Song *et al.* 2017). At meanwhile, the ECM composition interacts with VICs and provides binding sites for calcium (Rodriguez and Masters 2009). However, the composition of ECM is complex, and the regulatory mechanism of VICs osteogenic differentiation is not fully clarified. SIBLING family proteins, including DMP-1, osteopontin, bone sialoprotein, matrix extracellular phosphoglycoprotein, and dentin sialophosphoprotein, play important roles in the development of bone and teeth. Recently, their involvement in cardiovascular calcification has attracted growing attention. Notably, osteopontin has been reported to predict the prognosis of CAVD patients undergoing transcatheter aortic valve implantation (TAVI) (Lutz et al., 2017). Piotr Mazur *et al.* found that BSP-2 was positively associated with the severity of calcification (Mazur *et al.* 2017). Studies confirmed the expression of DMP-1 in vascular smooth muscle cells, but its role in valvular calcification was unclear (Zhu *et al.* 2011).

We found increased expression of DMP-1 in calcified valves compared with normal valves. The level of DMP-1 was positively correlated with RUNX2 and ALP, which were associated with the severity of calcification. There are several phosphorylation sites at DMP-1’s N-terminus that can bind with Ca2 + and act as the core of calcification (Deshpande *et al.* 2011). Besides, the acidic groups at the N-terminal of DMP-1 can bind with type I collagen, resulting in two different states of DMP-1 in vivo (Ustriyana *et al.* 2022). The RGD domain at the C-terminal of DMP-1 can interact with the integrin receptor (Pawade *et al.* 2015). We utilized a long RGD peptide, which resembles collagen-bound DMP-1, and a short RGD peptide, which emulates the dissociative form of DMP-1, to stimulate VICs.

Results demonstrated that both the long and short RGD peptides were capable of inducing osteogenic differentiation in VICs. In our study, we employed Cyclo (RGDyK), a compound with a binding affinity to integrin αvβ3 that is 10–100 times higher than that of the RGD peptide (Shimaoka and Springer 2003; Mas-Moruno *et al.* 2010), to inhibit the interaction between the RGD peptide and the integrin αvβ3 receptor. Results demonstrated that the antagonist could prevent the osteogenic differentiation of VICs. Therefore, the pro-osteogenic effect of DMP-1 might be mediated by the integrin αvβ3 receptor.

Our data revealed that β3 subunit can activate protein kinase and promote osteogenic gene expression via the MAPK signaling pathway. However, several studies demonstrated that RUNX2 expression regulation via other signaling pathways such as parathyroid hormone (PTH/PTHR), MAPK, SMAD, or NF-κB signaling pathway (Franceschi and Xiao 2003; Chen and Simmons 2011; Song *et al.* 2017; Zheng *et al.* 2022) indicated that integrin receptor is known to activate the expression of RUNX2 mainly through the MAPK pathway and our data further confirm this conclusion (Jiang and Tang 2018). But whether other signaling pathways participate in DMP-1-mediated valvular calcification still needs further investigation. The present study investigated the pro-osteogenic effect of DMP-1 and gave evidence that DMP-1 and integrin receptor could be therapeutic targets for CAVD. To further confirm the role of endogenous DMP-1 in valvular calcification, we silenced DMP-1 in VICs using siRNA under osteogenic induction. The results demonstrated that knockdown of DMP-1 significantly reduced ALP and RUNX2 expression and suppressed calcium nodule formation, suggesting that DMP-1 is essential for VICs to undergo osteogenic differentiation under calcifying conditions. In addition, we found that inhibition of integrin αvβ3 using cyclo-RGD similarly reduced calcification and downregulated osteogenic markers. Notably, combined treatment with si-DMP1 and αvβ3 antagonist produced a greater inhibitory effect than either treatment alone, implying that DMP-1 may act synergistically with integrin αvβ3 in promoting osteogenic signaling in VICs. These findings provide further support that both DMP-1 and integrin αvβ3 are critical mediators in the pathogenesis of aortic valve calcification and may represent combinational therapeutic targets for CAVD.

A limitation of our study is the small sample size, which restricts the generalizability of our findings on serum DMP-1 levels in patients. Future studies with larger cohorts are needed to confirm these preliminary findings and explore additional therapeutic approaches for CAVD.

## Conclusion

In our study, we found that the expression of DMP-1 was significantly increased in calcified aortic valves, which could bind to integrin αvβ3 via its RGD domain, promoting the osteogenic differentiation of VICs. Furthermore, we discovered that activation of the MAPK signaling pathway by DMP-1 led to elevated osteogenic gene expression in VICs. Our data indicated that DMP-1 is an important mediator of osteogenic transformation in VICs, which might be a potential target for treatment of CAVD.

## Supplementary Information

Below is the link to the electronic supplementary material.

Supplementary file1 (JPG 1913 KB)

Supplementary file2 (JPG 2537 KB)

## Data Availability

The data that support the findings of this study are available from the corresponding authors upon reasonable request.

## Abbreviations

*CAVD*: Calcific aortic valve disease
*DMP-1*: Dentin matrix protein-1
*ECM*: Extracellular matrix
*EF*: Ejection fraction
*LDL-C*: Low-density lipoprotein-cholesterol
*RUNX2*: Runt-related transcription factor 2
*RGD*: Arginine-glycine-aspartic acid
*VECs*: Valvular endothelial cells
*VICs*: Valvular interstitial cells
