# Supplementary figures and images for: Dentin matrix protein-1 promoted osteogenic differentiation of valvular interstitial cells via MAPK signal pathway during aortic valve calcification
Source: In Vitro Cell Dev Biol Anim. 2025 Oct 13;61(9):1057–70. doi: 10.1007/s11626-025-01101-7 (PMC12628416; doi:10.1007/s11626-025-01101-7)

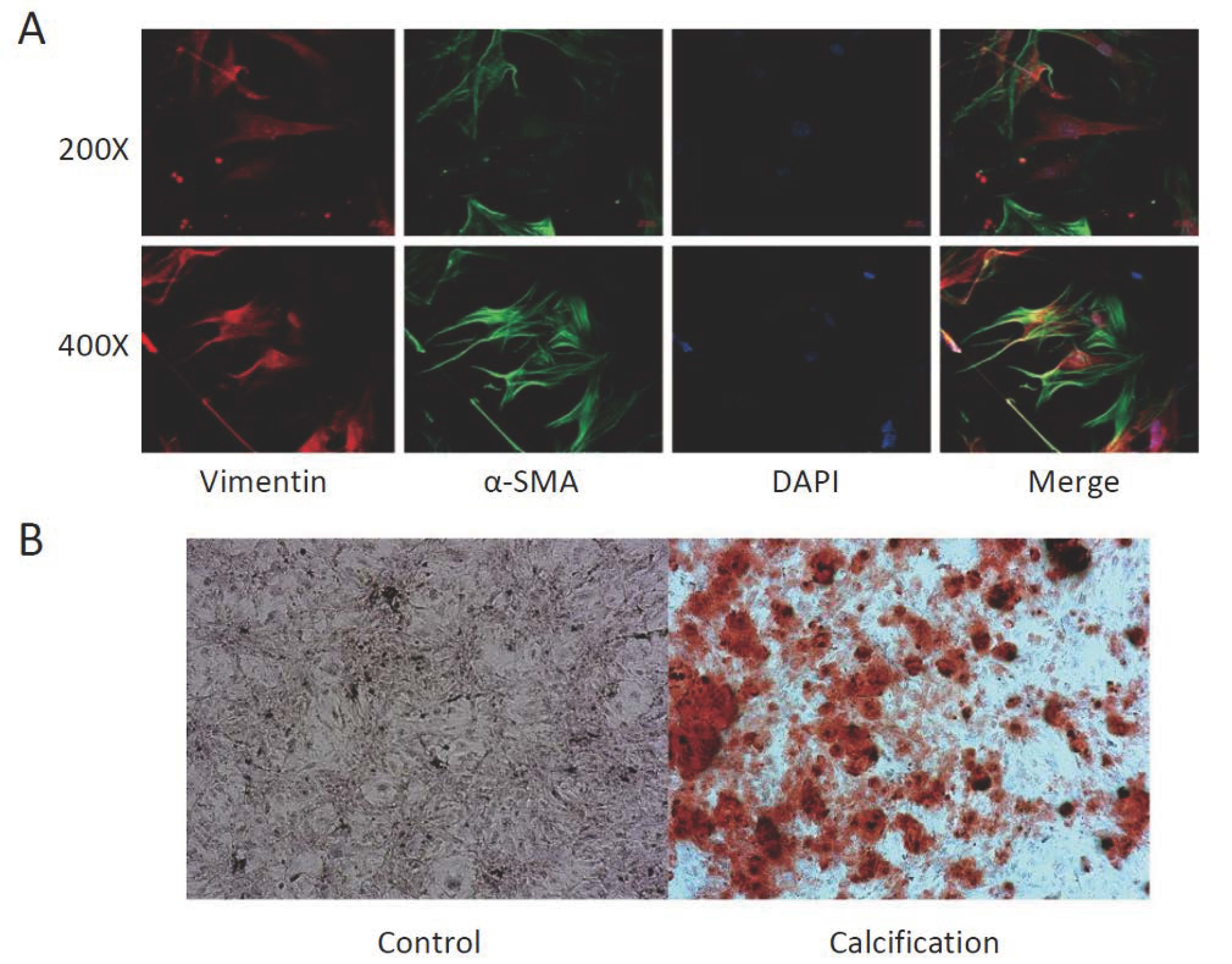

Supplement: Supplementary file 1 — Supplementary file1 (JPG 1913 KB) [file 11626_2025_1101_MOESM1_ESM.jpg]
